# Emergence and evolution of internationally disseminated cephalosporin-resistant *Neisseria gonorrhoeae* clones from 1995 to 2005 in Japan

**DOI:** 10.1186/s12879-015-1110-x

**Published:** 2015-09-17

**Authors:** Ken Shimuta, Yuko Watanabe, Shu-ichi Nakayama, Tomoko Morita-Ishihara, Toshiro Kuroki, Magnus Unemo, Makoto Ohnishi

**Affiliations:** National Institute of Infectious Diseases, Tokyo, Japan; Kanagawa Prefectural Institute of Public Health, Kanagawa, Japan; WHO Collaborating Centre for Gonorrhoea and other STIs, Department of Laboratory Medicine, Microbiology, Faculty of Medicine and Health, Örebro University, Örebro, Sweden; Department of Bacteriology I, National Institute of Infectious Diseases, 1-23-1 Toyama, Shinjuku, Tokyo 162-8640 Japan

## Abstract

**Background:**

*Neisseria gonorrhoeae* strains with resistance to extended-spectrum cephalosporins (ESCs), last options for first-line monotherapy of gonorrhoea, likely emerged and initially disseminated in Japan, followed by international transmission. In recent years, multi-locus sequence typing (MLST) ST1901 and *N. gonorrhoeae* multiantigen sequence typing (NG-MAST) ST1407 isolates with the mosaic penicillin-binding protein (PBP) 2 XXXIV have accounted for most ESC resistance globally. Our aim was to elucidate the initial emergence and transmission of ESC-resistant strains by detailed examination of *N. gonorrhoeae* isolates from 1995 to 2005 in Kanagawa, Japan.

**Methods:**

*N. gonorrhoeae* isolates were examined phenotypically (*n* = 690) and genetically (*n* = 372) by agar dilution method (cefixime, ceftriaxone and ciprofloxacin), *penA* gene sequencing, MLST and NG-MAST.

**Results:**

Already in 1995, one cefixime-resistant (CFM-R) isolate was found, which is the first CFM-R isolate described globally. After 1996, the prevalence of CFM-R and CFM-decreased susceptibility (CFM-DS) isolates significantly increased, with the peak resistance level in 2002 (57.1 % CFM-R). In 1997–2002, the CFM-R MLST ST7363 strain type with the mosaic PBP 2 X was predominant among CFM-R/DS isolates. The first CFM-R/DS MLST ST1901 clone(s), which became the predominant CFM-R/DS strain type(s) already in 2003–2005, possessed the mosaic PBP 2 X, which was possibly originally transferred from the MLST ST7363 strains, and subsequently acquired the mosaic PBP 2 XXXIV. The first MLST ST1901 and NG-MAST ST1407 isolate was identified in Kanagawa already in 2003.

**Conclusions:**

The two main internationally spread cefixime-resistant gonococcal clones, MLST ST7363 and ST1901 (NG-MAST ST1407 most frequent internationally) that also have shown their capacity to develop high-level ceftriaxone resistance (superbugs H041 and F89), likely emerged, evolved and started to disseminate in the metropolitan area, including Kanagawa, in Japan, which was followed by global transmission.

## Background

Gonorrhoea, aetiological agent *Neisseria gonorrhoeae*, is a major public health concern worldwide, and in 2008 the World Health Organization (WHO) estimated 106 million new cases among adults globally [1]. Since the introduction of antimicrobials for treatment of gonorrhoea in the mid-1930s, *N. gonorrhoeae* has developed resistance to all previously used first-line antimicrobials for empirical treatment of gonorrhoea. In many countries, the extended-spectrum cephalosporins (ESCs) are the only remaining options for empirical antimicrobial monotherapy [2–7]. Disquietingly, treatment failures with cefixime (CFM) have been reported in several countries [3, 8, 9], and rare cases of treatment failures with the more potent ceftriaxone (CRO) have been verified in a few countries [3, 7, 10–12]. Most of these ESC treatment failures have been caused by multidrug resistant (MDR) *N. gonorrhoeae* strains belonging to the multi-locus sequence typing (MLST) sequence types (ST) ST7363 and ST1901 [3, 4, 7, 9, 12, 13]. Among the ESC-resistant MLST ST1901 gonococcal strains, isolates assigned to the *N. gonorrhoeae* multiantigen sequence typing (NG-MAST) ST1407 have been the most frequent [3, 4, 8, 9, 12]. Furthermore, MLST ST7363 (NG-MAST ST4220) and MLST ST1901 (NG-MAST ST1407) strains have also shown their capacity to develop high-level *in vitro* resistance to CRO. These types of strains, which has been referred to as “superbugs”, were isolated in Japan (“H041”), France (“F89”) and Spain (“F89”) from 2009 to 2011 [7, 14, 15], which might represent the initial phase of dissemination of gonococcal strains with high-level CRO resistance. The main ESC-resistance mechanism in these high-level CRO-resistant gonococcal isolates was novel mosaic *penA* gene alleles, encoding the ESC-target penicillin-binding protein 2 (PBP 2) [3, 7, 14, 15]. ESC-resistant MLST ST7363 and ST1901 *N. gonorrhoeae* strains have been frequently associated with the mosaic PBP 2 X and XXXIV, respectively [3, 4, 7, 9, 12, 13].

Over the past two decades, gonococcal strains with decreased susceptibility and resistance to ESCs have been suspected to have emerged in Japan and subsequently spread internationally [2–4]. For example, from 1995 to 2000, in Fukuoka, Japan, the MIC peak of CFM and CRO in gonococcal isolates increased from 0.008 mg/L to 0.25 mg/L and from 0.015 mg/L to 0.064 mg/L, respectively [16]. Furthermore, from 1999 to 2002 in six hospitals in central Japan, the proportion of gonococcal isolates with decreased susceptibility or resistance to CFM (MIC ≥ 0.5 mg/L) and CRO (MIC ≥ 0.5 mg/L) increased from 0 % to 30.2 % and from 0 % to 0.9 %, respectively [17]. The first treatment failures with CFM were also described in Japan, i.e. in the late 1990s and early 2000s [18, 19]. Accordingly, already in the late 1990s ESC-resistant *N. gonorrhoeae* strains emerged and were disseminated in Japan. However, the genetic relatedness between the ESC-resistant *N. gonorrhoeae* strains spreading in Japan 1–2 decades ago and the ESC-resistant gonococcal strains currently spreading internationally has not been investigated appropriately. It is crucial to understand the emergence and spread of ESC- and MDR-resistant gonococcal strains globally in order to develop and implement evidence-based strategies for prevention and control of gonorrhoea.

The aim of this study was to examine 690 (372 genetically) *N. gonorrhoeae* isolates cultured from 1995 to 2005 in Japan (the initial phase of emergence and dissemination of ESC-resistant strains) using antimicrobial susceptibility testing, *penA* gene sequencing, MLST and NG-MAST.

## Methods

### *Neisseria gonorrhoeae* isolates

In total, 690 *N. gonorrhoeae* isolates, cultured at the Kanagawa Prefectural Institute of Public Health, Japan from 1995 to 2005, were included. *N. gonorrhoeae* isolates were cultured, species identified and stored as described previously [20, 21]. All examined gonococcal isolates were cultured and preserved as part of the routine diagnostics (standard care) and no patient identification information was available in the present study. Accordingly, ethical approval was not required for this study.

### Antimicrobial susceptibility testing

The MICs (mg/L) of CFM, CRO and ciprofloxacin were determined for all isolates immediately after initial isolation by the agar dilution method, according to the instructions from the Clinical and Laboratory Standards Institute (CLSI) [22]. However, the MIC values for all the isolates with resistance (R) or decreased susceptibility (DS) to CFM (CFM-R/DS) were also verified in the present study by repeated testing using the identical agar dilution method. The resistance breakpoints stated by the European Committee on Antimicrobial Susceptibility Testing (EUCAST; www.eucast.org) were applied. However, additionally CFM-DS and CRO-DS was defined as MIC = 0.125 mg/L, because treatment failures with both CFM and CRO have been caused by gonococcal isolates with this MIC [3, 8, 10–12]. The *N. gonorrhoeae* international reference strains WHO A, B and E were used for quality control.

### DNA extraction

The bacterial isolates were suspended in TE buffer (10 mM Tris, 1 mM EDTA, pH 8.0) and boiled for 10 min. After centrifugation to remove cell debris, the supernatant was promptly used as DNA template for the PCRs.

### *penA* gene sequencing

The *penA* gene was PCR amplified and sequenced using the previously described primers penA_F and penA_R [13]. Briefly, the PCR mixtures were incubated for 2 min at 96 °C, followed by 30 cycles of 10 s at 96 °C, 10 s at 65 °C and 2 min at 72 °C. The PCR products were subsequently purified with the ExoSAP IT kit (GE Healthcare Limited, Buckinghamshire, United Kingdom). Both DNA strands of the PCR products were sequenced with an ABI BigDye Terminator cycle sequencing kit (version 3.1) on an ABI 3130 xl sequencer, in accordance with the instructions from the manufacturer (Applied Biosystems, Foster City, CA, USA). The *penA* gene that encodes the PBP 2 X sequence variant has a mosaic-like structure that includes regions relatively similar to the corresponding regions of the *penA* genes of *N. perflava*, *N. sicca* and *N. cinerea* [23]*.* Subsequently, many different mosaic PBP 2 sequences have been described, which have up to 60 to 70 amino acid changes compared to a wild type PBP 2 sequence [7]. In the present study, all these sequences are named as mosaic PBP 2 sequence variants and wild type PBP 2 (e.g., GenBank accession no. M32091) and highly similar sequences are defined as non-mosaic PBP 2 sequences.

The nucleotide sequences of *penA* determined in the present study have been deposited in the DDBJ sequence library and assigned the accession numbers LC055782 and LC055783.

### Molecular epidemiological characterisation

Molecular epidemiological characterisation by means of MLST (based on partial alleles of seven loci, *abcZ, adk, aroE, fumC, gdh, pdhC* and *pgm*) and NG-MAST (based on partial alleles of *porB* and *tbpB*) was performed as described elsewhere [24]. New alleles (and STs) identified in this study were named and deposited in the MLST and NG-MAST database, respectively (Tables 1 and 2). The diversity index for the MLST and NG-MAST was calculated as described earlier [24]. UPGMA trees based on partial *porB* gene sequences (490 bp), i.e. obtained in the NG-MAST, were generated using the MEGA 4 software.

**Table 1:** New multi-locus sequence typing (MLST) sequence types (STs) identified in this study

MLST ST	*abcZ*	*adk*	*aroE*	*fumC*	*gdh*	*pdhC*	*pgm*	Representative strain	
								Strain name	Isolated year
10622	59	39	67	156	149	153	133	NG9837	1998
10623	59	39	67	111	682^a^	153	133	NG9845	1998
10624	59	39	67	111	150	153	133	NG9930	1999
10625	109	39	170	156	150	71	65	NG9931	1999
10626	126	39	67	159	149	154	65	NG9943	1999
10627	109	39	67	156	149	71	65	NG9942	1999
10628	59	39	170	159	188	71	65	NG9934	1999
10629	660^a^	39	170	78	147	153	65	NG9918	1999
10630	660^a^	39	67	78	147	153	65	NG9919	1999
10631	109	39	170	111	152	153	65	NG0168	2001
10632	109	39	170	156	188	153	65	NG0177	2001
10633	126	39	170	78	147	153	65	NG0121	2001
10634	59	39	67	78	149	153	65	NG0157	2001
11052	59	39	754^a^	78	148	153	65	NG0330	2003

^a^New alleles

**Table 2:** New *Neisseria gonorrhoeae* multiantigen sequence typing (NG-MAST) sequence types (STs) identified in this study

NG-MAST	*porB*	*tbpB*	Representative strain	
			Strain name	Isolated year
9027	2494	856	NG9858	1998
9028	1056	241	NG9850	1998
9029	3599	241	NG9860	1998
9030	916	241	NG9870	1998
9031	2296	24	NG9874	1998
9032	4424	10	NG9816	1998
9033	854	10	NG9880	1998
9034	2742	4	NG9882	1998
9035	3986	361	NG9883	1998
9036	4226	10	NG9828	1998
9038	161	137	NG9845	1998
9039	5349^a^	241	NG0177	2001
9040	5347^a^	10	NG0178	2001
9041	5348^a^	241	NG0173	2001
9042	5350^a^	241	NG0174	2001
9043	5346^a^	110	NG0115	2001
9044	444	873	NG0216	2002
9045	566	27	NG0211	2002
9046	673	32	NG0214	2002
11434	2461	19	NG0346	2003
11436	5186	113	NG0469	2004
11437	2522	113	NG0467	2004
11438	4260	10	NG0460	2004
11439	6705^a^	10	NG0324	2003
11440	6710^a^	10	NG0325	2003
11441	6718^a^	10	NG0327	2003
11442	6716^a^	10	NG0328	2003
11443	6712^a^	10	NG0330	2003
11444	6715^a^	10	NG0334	2003
11445	6701^a^	110	NG0335	2003
11446	6706^a^	21	NG0340	2003
11447	6707^a^	276	NG0342	2003
11448	6699^a^	10	NG0344	2003
11449	6702^a^	10	NG0353	2003
11450	6704^a^	4	NG0425	2004
11451	6700^a^	29	NG0426	2004
11452	6714^a^	110	NG0435	2004
11453	6708^a^	10	NG0437	2004
11454	6703^a^	10	NG0440	2004
11455	6717^a^	110	NG0442	2004
11456	6709^a^	10	NG0448	2004
11457	6713^a^	241	NG0465	2004
11458	6711^a^	29	NG0466	2004

^a^New alleles

### Statistical analysis

The distribution of MLST ST7363 in CFM-S and CFM-R/DS strains was analysed by Fisher’s exact test.

## Results

### Emergence of cephalosporin resistance in *Neisseria gonorrhoeae* in Japan

Already in 1995, among 34 *N. gonorrhoeae* isolates one CFM-R isolate (MIC = 0.25 mg/L), which is the first CFM-R isolate ever described, and one CFM-DS isolate (MIC = 0.125 mg/L), were identified. No CFM-R/DS isolates were found in 1996. However, from 1997 (one CFM-R isolate with MIC = 0.25 mg/L) and onwards, the prevalence of CFM-R and CFM-DS isolates significantly increased, with the peak resistance level in 2002, where 57.1 % of the examined isolates were CFM-R. The first isolate with CRO-DS was identified in 2000. CRO-DS isolates were subsequently found annually in 2001–2005, with a peak prevalence of 29.1 % in 2003 (Table 3).

**Table 3:** Resistance and decreased susceptibility to cefixime (CFM) or ceftriaxone (CRO) in *Neisseria gonorrhoeae* isolates cultured from 1995 to 2005 in Kanagawa area, Japan

Year	Number of isolates^a^	CFM-DS^a,b^		CFM-R^a,c^		CRO-DS^a,b^	
		No.^a^	%	No.^a^	%	No.^a^	%
1995	34 (1)	1 (1)	2.9	1 (0)	2.9	0	0
1996	70 (0)	0	0	0	0	0	0
1997	88 (1)	0	0	1 (1)	1.1	0	0
1998	89 (84)	0	0	5 (5)	5.6	0	0
1999	69 (50)	2 (2)	2.9	3 (3)	4.3	0	0
2000	54 (49)	6 (5)	11.1	6 (5)	11.1	1 (1)	1.9
2001	102 (90)	10 (9)	9.8	34 (32)	33.3	17 (17)	16.7
2002	21 (21)	0	0	12 (12)	57.1	4 (4)	19.0
2003	55 (27)	5 (4)	9.1	26 (23)	47.3	16 (14)	29.1
2004	74 (34)	10 (10)	13.5	29 (24)	39.2	16 (13)	21.6
2005	34 (15)	8 (6)	23.5	10 (9)	29.4	5 (5)	14.7

^a^Number of isolates examined genetically is given in parentheses

^b^MIC = 0.125 mg/L of cefixime or ceftriaxone

^c^MIC > 0.125 mg/L of cefixime

### *penA* (encoding PBP 2) sequences of CFM-R/DS *Neisseria gonorrhoeae* isolates in Japan

The CFM-R isolate from 1995 was not possible to recover. However, the CFM-DS isolate from 1995 contained the non-mosaic PBP 2 XIII [7, 25], whereas the CFM-R isolate from 1997 possessed the mosaic PBP 2 X [3, 7, 13]. These two isolates from 1995 to 1997 were considered the original CFM-R/DS isolates in the present study.

**Fig. 1:**
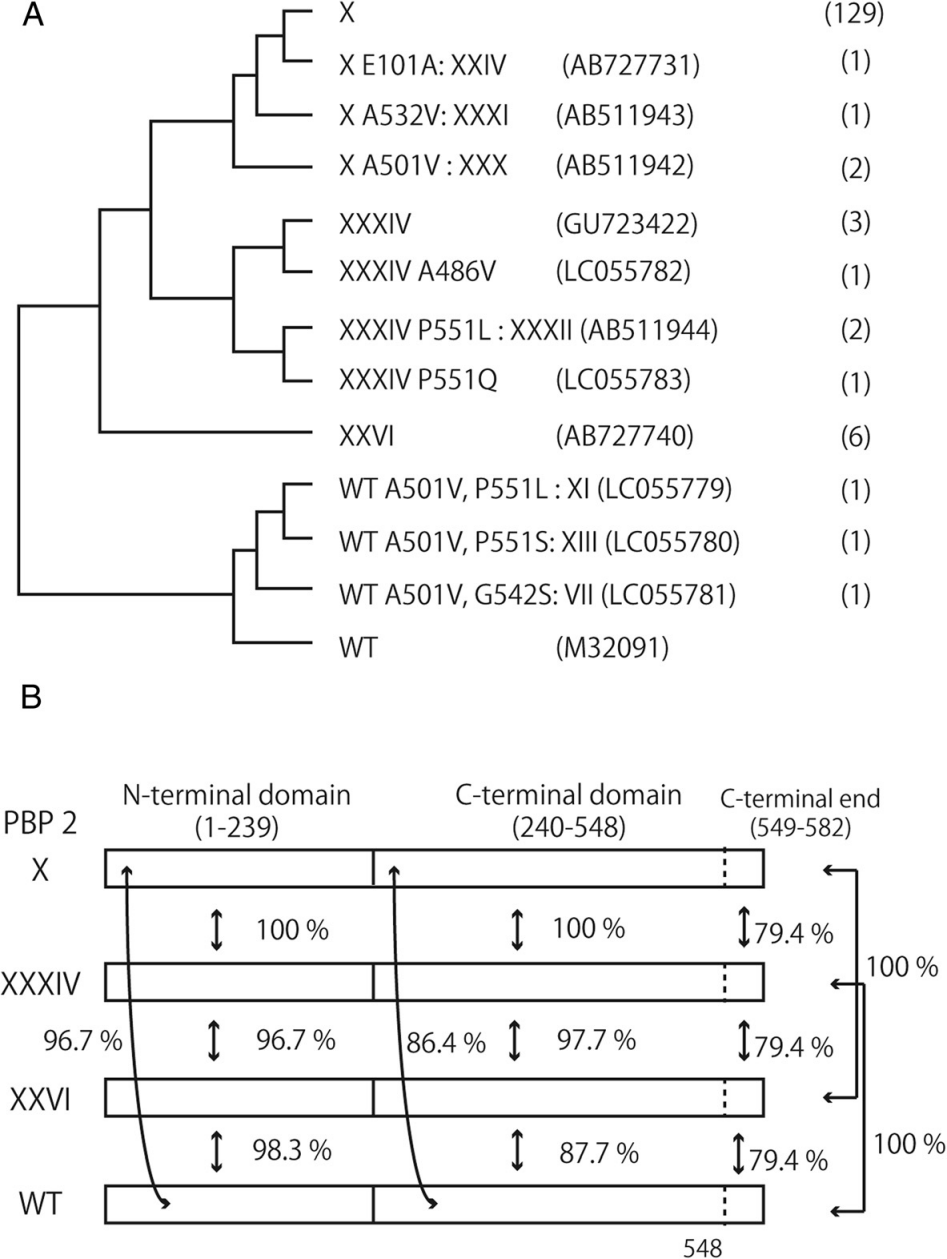
*Neisseria gonorrhoeae* penicillin-binding protein 2 (PBP 2) amino acid sequences in strains with resistance or decreased susceptible to cefixime. **a** The dendrogram analysis of amino acid sequences included 12 PBP 2 sequences from *N. gonorrhoeae* with resistance and decreased susceptibility to cefixime and a wild-type (WT) PBP 2 sequence (M32091). Lower half contains the wild-type PBP 2 sequence and amino acid alterations in the non-mosaic PBP2 XI, XIII and VII, which possessed two amino acid substitutions compared to WT. Upper half displays the mosaic PBP 2 X, XXXI and XXXIV and their single amino acid variants that were found in this study. The numbers of isolates in this study are shown in parentheses. **b** Amino acid sequence similarities of mosaic PBP 2 (X, XXXIV and XXVI) and WT are shown. The boundary of N- and C-terminal domain is from the crystal structure of PBP 2 derived from the penicillin-resistant strain FA19 [33]. The N-terminal domain (1–239) of the mosaic PBP 2 sequences are similar to WT (over 96.7 %), but the C-terminal domains of mosaic PBP 2 show lower similarity: 86.4 % for PBP 2 X and PBP 2 XXXIV and 87.7 % for XXVI compared to WT. PBP 2 X is identical to PBP 2 XXXIV, except for the C-terminal end (549–582, where seven amino acids differ). The C-terminal end of PBP 2 XXXIV is identical with that of WT, whereas that of PBP 2 X is identical to PBP 2 XXVI, although the C-terminal domain of PBP 2 XXVI differs from the mosaic PBP 2 X and XXXIV (97.7 % identity). For detailed amino acid sequences of these and other PBP 2’s, see Ohnishi et al. [7]

Among the CFM-R and CFM-DS isolates from 1998 to 2005 (*n* = 166), 149 (90 %) isolates were possible to analyse genetically; of these 149, 12 PBP 2 amino acid sequence variants were found. Nine were mosaic PBP 2 and three were non-mosaic PBP 2 sequence variants (VII, XI, XIII). The most prevalent sequence type was the mosaic PBP 2 X (86.6 %, 129/149). The additionally identified mosaic PBP 2 sequence variants were variants of X, i.e. with single amino acid substitutions (XXIV, XXX, XXXI), XXVI, XXXIV, and three XXXIV variants with single amino acid substitutions (Fig. 1). Most noteworthy, the first CFM-DS isolates with the mosaic PBP 2 XXXIV (*n* = 2) were identified already in 2001. All the non-mosaic PBP 2 sequence variants contained the A501V alteration and additionally alterations in G542 or P551 (Fig. 1, Table 4).

**Table 4:** Multilocus sequence typing (MLST) and penicillin-binding protein 2 (PBP 2) variants [7] in *Neisseria gonorrhoeae* isolates (*n* = 149) cultured from 1998 to 2005 (only isolates with resistance or decreased susceptibility to cefixime) in Kanagawa area, Japan

		PBP 2 X family				PBP 2 XXXIV family							
MLST	n =	X	+ E101D (XXIV)	+ A501V (XXX)	+ A532V (XXXI)	XXXIV	+ P551L (XXXII)	+A486V	+ P551Q	XXVI	VII	XI	XIII
7363	99	93		2		3		1					
1901	14	9	1				2		1				1
1596	13	13											
7358	6									6			
1588	2	2											
1590	2				1						1		
7371	2	2											
11052	2	2											
1579	1	1											
1600	1	1											
7356	1	1											
7367	1	1											
7827	1	1											
8153	1	1											
10631	1	1											
10633	1											1	
10634	1	1											

### MLST analysis of CFM-R/DS *Neisseria gonorrhoeae* isolates in Japan

The CFM-DS isolate from 1995, with the non-mosaic PBP 2 XIII [7, 25], was assigned to MLST ST7365, whereas the CFM-R isolate from 1997 (mosaic PBP 2 X [3, 7, 13]) belonged to MLST ST7363.

**Fig. 2:**
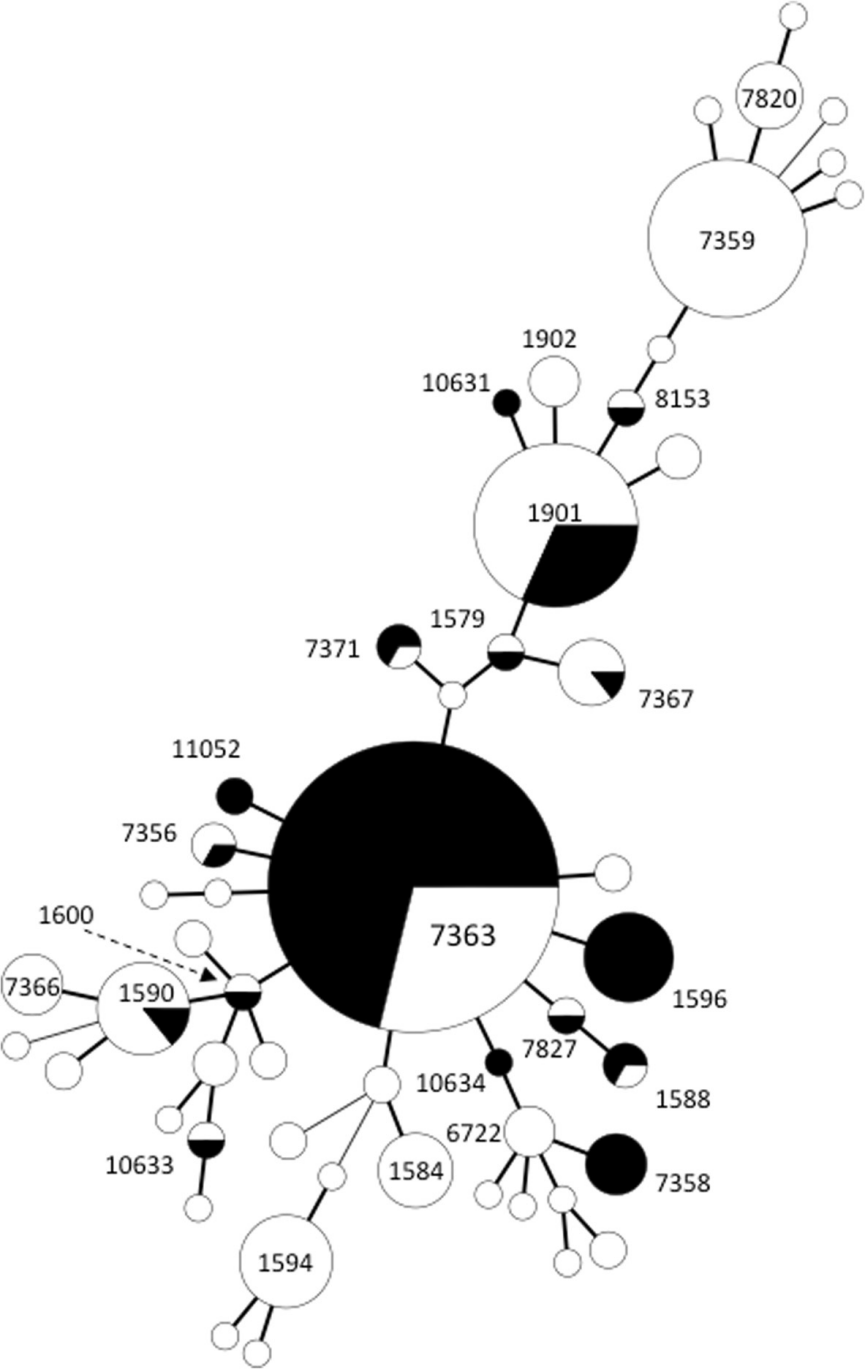
Molecular epidemiological relatedness of *Neisseria gonorrhoeae* isolates from 1998 to 2005 in the Kanagawa area, Japan. Minimal spanning tree of 48 MLST STs shows genetic distance of STs derived from 370 isolates. Circle sizes denote the number of isolates sharing the same ST. Black indicates *N. gonorrhoeae* isolates with resistance or decreased susceptibility to cefixime

In total, 370 isolates from 1998 to 2005 (only CFM-R/DS isolates from 2003 to 2005) were analysed using MLST; all CFM-R/DS isolates are summarised in Table 4. As shown in Fig. 2, among all isolates 52 MLST types were revealed (diversity index = 0.825). Despite that the CFM-R/DS isolates belonged to 17 MLST STs, the diversity index of the CFM-R/DS isolates (DI = 0.539) was substantially lower than that of CFM-S isolates (DI = 0.901).

During 1998–2002, MLST ST7363 was the most prevalent ST in both CFM-S (40/221, 19.9 %) and CFM-R/DS strains (53/73, 72.6 %), but the proportion was significantly higher in the CFM-R/DS isolates (*P* < 0.01). During 2003–2005, 61.3 % (46/76) of the CFM-R/DS isolates were assigned as MLST ST7363. During 1998–2005, all except six CFM-R/DS ST7363 isolates possessed the mosaic PBP 2 X. The remaining six isolates contained the mosaic PBP 2 XXXIV (*n* = 3), XXX (X-A501V; *n* = 2) and XXXIV-A486V (*n* = 1), indicating an evolution of PBP 2 X or that the new PBP 2 mosaic structures developed during the dissemination of the CFM-R/DS ST7363 strains. However, 50 of the CFM-R/DS isolates were assigned as other MLST STs (*n* = 16) than ST7363. Six of these 16 MLST STs (ST1596, ST1600, ST7356, ST7827, ST10634 and ST11052) identified in 19 isolates were single locus variants of ST7363. All these 19 isolates had the mosaic PBP 2 X. Another putative MLST ST cluster that included CFM-R/DS isolates consisted of ST1579 (*n* = 1), ST1901 (*n* = 14), ST8153 (*n* = 1) and ST10631 (*n* = 1), which differed from ST7363 at 2, 3, 4 and 4 loci, respectively, suggesting that ST1901 and its single locus variants ST1579, ST8153 and ST10631 belong to a genetically different group from ST7363. Nevertheless, 12 (71 %) of these 17 isolates also possessed the mosaic PBP 2 X. Particularly noteworthy is that the proportion of MLST ST1901 isolates among the CFM-R/DS isolates significantly increased from 2.6 % (2/78) in 1998–2002 to 15.8 % (12/76) in 2003–2005. Furthermore, during 2003–2005, all three isolates possessing XXXIV or XXXIV-related PBP 2 sequences were assigned to MLST ST1901 (Table 4). One of these isolates (from 2003) was also assigned as NG-MAST ST1407 (*porB908* and *tbp110*). This is the first isolate described worldwide of this MDR clone that has subsequently accounted for most of the reported ESC resistance globally [3, 4, 8, 9, 12, 14, 15, 24, 26].

### NG-MAST analysis of CFM-R/DS *Neisseria gonorrhoeae* isolates in Japan

As described above, isolates assigned as MLST ST7363 and containing the mosaic PBP 2 X were predominant among the CFM-R/DS isolates from 1998 to 2005. To further investigate the clonality of all the CFM-R/DS strains from 1995 to 2005 NG-MAST analysis was applied for the same 372 isolates (only CFM-R/DS isolates from 2003 to 2005). NG-MAST analysis revealed 194 STs and a high DI (0.988). Eighty-seven STs were identified among the CFM-R/DS isolates (DI: 0.976), showing that the CFM-R/DS strains have further evolved.

The CFM-DS isolate from 1995 was assigned as the NG-MAST ST4045 (*porB2445* and *tbpB29*), which was not subsequently identified during 1997–2005. The CFM-R isolate from 1997 belonged to the NG-MAST ST4127 (*porB2520* and *tbpB10*), which two CFM-R isolates were also assigned to in 1998. However, ST4127 isolates, even isolates with *porB2520*, were not found after 1998.

**Fig. 3:**
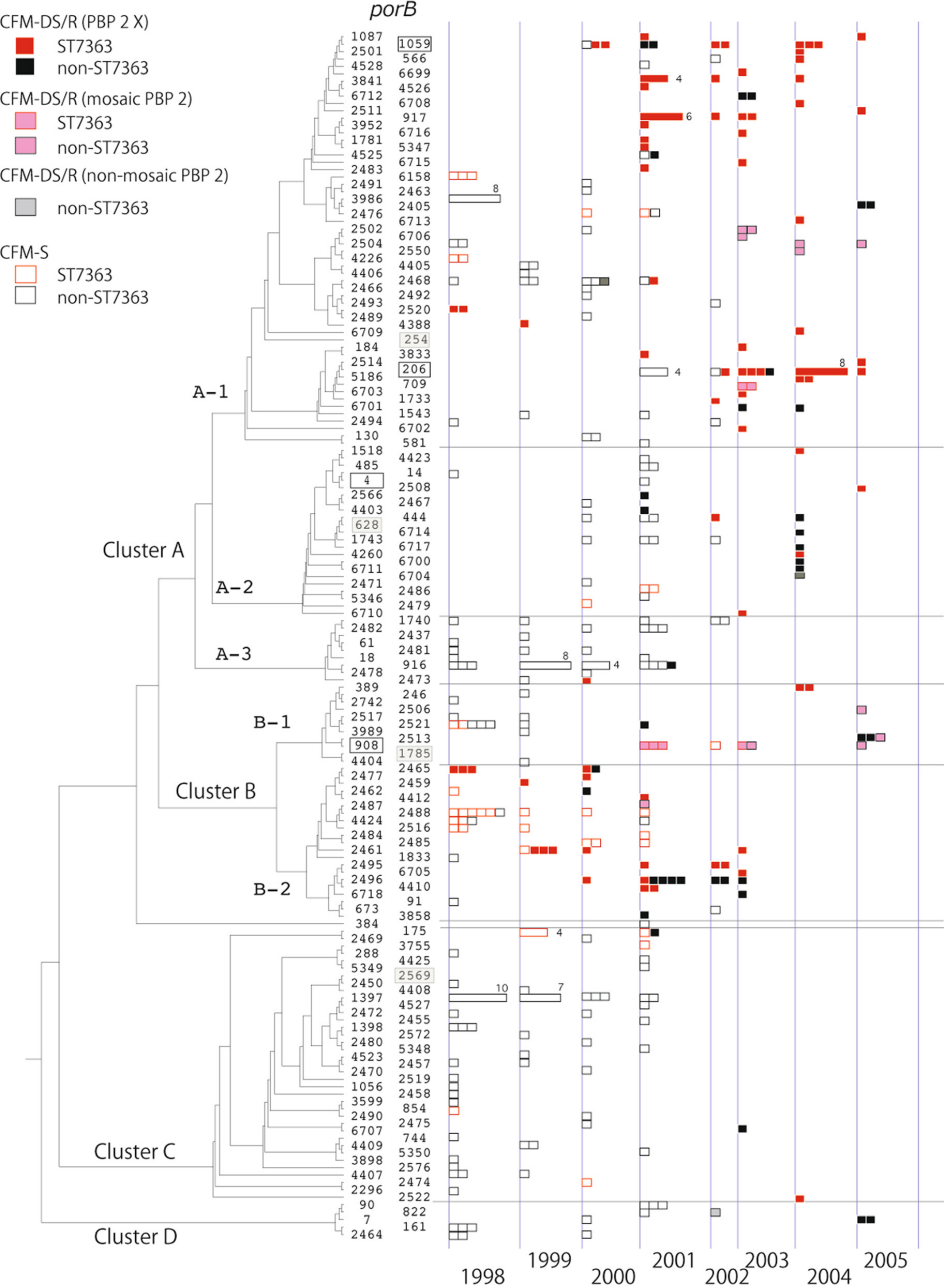
Comparison of the *porB* gene sequences in *Neisseria gonorrhoeae* isolates obtained from 1998 to 2005 in the Kanagawa area, Japan. Dendrogram was constructed using 163 partial *porB* sequences (490 bp) by UPGMA. One -hundred -fifty-nine *porB* sequences were from *N. gonorrhoeae* isolates examined in this study, including four of the major *porB* alleles in the Kyoto/Osaka strains isolated during 2010–2012 [24], (depicted in the shaded frame). The two remaining of the major *porB* alleles in the Kyoto/Osaka strains (*porB1785* and *porB2569*) [24] and *porB254* and *porB628*, from strains with cefixime resistance in Sweden (2002) and the USA (2003) [27], were also included with the *porB* type framed. Each box illustrates an individual strain. When more than four isolates with the identical *porB* type were cultured in the same year, the number of isolates is shown. Red and black boxes indicate MLST ST7363 strains and non-ST7363, respectively. Filled boxes indicate CFM-R/DS strains. Red and black filled rectangles, ST7363 and non-ST7363 strains with PBP 2 X, pink and gray are mosaic PBP 2 other than PBP 2 X and non-mosaic PBP 2

To describe to a great extent the evolution of the isolates examined in the present study the *porB* gene sequences in isolates from 1998 to 2005 (only CFM-R/DS isolates from 2003 to 2005; *n* = 370) were compared (Fig. 3). For comparison, the six major *porB* alleles from isolates cultured in Kyoto and Osaka from 2010 to 2012 (*porB4, porB206, porB908, porB1059, porB1785* and *porB2569*) [24] and *porB254* and *porB628*, which were reported in CFM-R isolates in Sweden and the USA in 2002 and 2003 [27], respectively, were also included in the analysis (Fig. 3). Of these eight *porB* alleles, four of the *porB* alleles from the Kyoto and Osaka collection (*porB4, porB206, porB908, porB1059*) were also found in the present study. Seven clusters were generated by the *porB* sequences: clusters (CL) A-1, A-2, A-3, B-1, B-2, C and D (Fig. 3; Table 5). The CL A-1 cluster was the largest cluster, containing 56 *porB* alleles from 132 isolates. The CL A-1 cluster included *porB2520*, which was from the first putative CFM-R isolates found in 1997 and 1998. Sixty-six (66.7 %) of the 99 MLST ST7363 CFM-R/DS isolates from 1998 to 2005 had one of the CL A-1 *porB* sequences and 64 (64.6 %) had the mosaic PBP 2 X. Furthermore, 64.5 % (49/76) of all the CFM-R/DS isolates from 2003 to 2005 belonged to the CL A-1 cluster. As mentioned above, the CFM-R/DS isolates with *porB2520* from 1997 to 1998 did not appear to be widely disseminated. Instead, isolates with other *porB* sequences, such as *porB1059*, *porB917* and *porB206* in the CL A-1 cluster, appeared to become dominant (Fig. 3).

**Table 5:** Penicillin-binding protein 2 (PBP 2) [7] and *porB* gene sequence cluster (A1-D) in *Neisseria gonorrhoeae* isolates (*n* = 149) cultured from 1998 to 2005 (only isolates with resistance or decreased susceptibility to cefixime in 2002–2005) in Kanagawa area, Japan

PBP 2 (2003–2005, *n* = 76^a^)		A1	A2	A3	B1	B2	C	D
X	X (62)	41	9	0	4	4	2	2
	+ E101D (XXIV) (1)				1			
	+ A501V (XXX) (2)	2						
XXXIV	XXXIV (1)				1			
	+ P551L (XXXII) (2)				2			
	+ P551Q (1)				1			
-	XXVI (6)	6						
PBP 2 (1998–2002, *n* = 73^b^)		A1	A2	A3	B1	B2	C	D
X	X (67)	33	3	2	1	27	1	0
	+ A532V (XXXI) (1)					1		
XXXIV	XXXIV (2)				2			
	+ A486V (1)				1			

^a^One isolate possessed the non-mosaic PBP 2 XIII [7]. The *porB* sequence from this isolate belonged to the cluster A2

^b^Two isolates possessed the non-mosaic PBP 2 VII and XI [7]

The CL B-2 cluster contained 21 *porB* gene sequences. *porB2465*, found in three CFM-R isolates in 1998, belonged to this *porB* sequence cluster. In 1998–2002, 18 (34.0 %) of the 53 CFM-R/DS MLST ST7363 isolates had *porB* gene sequences in the CL B-2 cluster. However, in 2003–2005 only two (4.3 %) of the 46 CFM-R/DS MLST ST7363 isolates belonged to the CL B-2 *porB* sequence cluster. In contrast to the CFM-R/DS MLST ST7363 isolates, *porB* sequences from non-ST7363 CFM-R/DS isolates with the mosaic PBP 2 X were found in all seven *porB* sequence clusters (Fig. 3).

Among the CFM-R/DS isolates with PBP 2 non-X sequence variants, isolates with the non-mosaic PBP 2 VII, XI and XIII possessed *porB2468* in CL A-1, *porB822* in CL E and *porB6704* in CL A-2, respectively. Four isolates with mosaic PBP 2 X-like sequence variants (XXIV (*n* = 1), XXX (*n* = 2) and XXXI (*n* = 1)) had three *porB* alleles: *porB908* (CL B-1), *porB709* (CL A-1) and *porB2467* (CL A-2), respectively. All seven CFM-R/DS isolates with the mosaic PBP 2 XXXIV, which is now the most prevalent mosaic PBP 2 sequence variant in CFM-R/DS isolates globally [3, 4, 8, 9, 12, 24, 26], or XXXIV-like sequence variants possessed *porB* alleles in the CL B-1, i.e. *porB908* (*n* = 5), *porB2516* (*n* = 1) and *porB2513* (*n* = 1). Among five CFM R/DS isolates possessing PBP 2 XXXIV or its relative with *porB908*, four belonged to MLST ST7363. However, most noteworthy is that the remaining isolate was assigned as MLST ST1901 and NG-MAST ST1407 (*porB908* and *tbpB110*) and contained the PBP 2 XXXIV with an additional P551Q mutation. The *por908* was, in general, the major *porB* of CFM-R/DS isolates belonging to the MLST ST1901. The MDR *N. gonorrhoeae* clone MLST ST1901, NG-MAST ST1407 with a PBP 2 mosaic XXXIV has accounted for most of the ESC resistance globally during the recent decade [3, 4, 8, 9, 12, 24, 26] and this clone has also shown its capacity to develop high-level ceftriaxone resistance, i.e., develop into a superbug such as F89 [14, 15].

## Discussion

Gonorrhoea and the high level of antimicrobial resistance in *N. gonorrhoeae* represent major public health concerns globally [1–6]. Treatment failures with the ESCs, the last remaining options for first-line empirical monotherapy of gonorrhoea, have been reported in several countries [3, 4, 7–12]. Most of these ESC treatment failures have been caused by MDR *N. gonorrhoeae* strains belonging to the MLST ST7363 and ST1901 [3, 4, 7, 9, 12, 13]. Among the ESC-resistant MLST ST1901 gonococcal strains, isolates assigned as the NG-MAST ST1407 have been the most frequent worldwide [3, 4, 8, 9, 12], and this clone has also shown its capacity to develop high-level ceftriaxone resistance, i.e., develop into a superbug such as F89 [14, 15].

In the present study the initial emergence and dissemination of these internationally transmitted ESC-resistant gonococcal clones in Japan from 1995 to 2005 were investigated. We showed that the first CFM-R isolate was cultured as early as 1995 in the Kanagawa area, Japan, which is four years earlier than previously recorded. This CFM-R isolate (MIC = 0.25 mg/L) possessed a non-mosaic PBP 2 XIII sequence variant [7, 25], with the amino acid alterations A501V and P551S that increase the ESC MICs [3, 4, 24, 28, 29]. This first CFM-R strain did not appear to spread widely; however, two years later (in 1997) one CFM-R MLST ST7363 isolate with the mosaic PBP 2 X [3, 7, 13] was found. During 1998–2002, this was the predominant CFM-R/DS strain type in the Kanagawa area, which is a neighbouring area of Tokyo. In contrast, in 1998–2002 MLST ST1901 isolates accounted for 11 % (32/294) of the isolates, but only two were CFM-R. Furthermore, no isolate was typed as NG-MAST ST1407 and the two CFM-R MLST ST1901 isolates possessed the mosaic PBP 2 X, and not the nowadays frequent mosaic PBP 2 XXXIV [3, 4, 8, 9, 12, 24, 26]. This observation indicates that the CFM-R/DS MLST ST1901 clone(s) were originally possessing the mosaic PBP 2 X, which possibly was originally transferred from the MLST ST7363 strains spreading widely, and subsequently, acquired the mosaic PBP 2 XXXIV that presently is the most frequent PBP 2 sequence variant in ESC-R/DS MLST ST1901 and NG-MAST ST1407 isolates [3, 4, 8, 9, 12, 24, 26]. In the present study it was also shown that the first MLST ST1901 and NG-MAST ST1407 isolate was cultured already in 2003. This is substantially earlier than the previously first described NG-MAST ST1407 strain, which possessed the mosaic PBP 2 XXXIV, identified in the USA in 2008 [30]. Since then, this strain type has been found to account for most of the ESC-R/DS isolates globally [3, 4, 8, 9, 12, 24, 26] and this clone has also shown its capacity to develop high-level ceftriaxone resistance, i.e., develop into a superbug such as F89 [14, 15]. All 11 CFM-R/DS isolates from 1997 to 1999 were assigned to MLST ST7363 (mosaic PBP 2 X), but the NG-MAST analysis revealed two major types of CFM-R/DS isolates, i.e. due to the diversification of the *porB* gene (*porB2520* in CL A-1 and *porB2465* in CL B-2). These two clones were considered to have emerged and started to disseminate in the Metropolitan area, including Kanagawa. Introduction from some other area(s) was highly unlikely because there is no recorded isolation of CFM-R gonococcal strains before 1999 in any other place. The reasons for the initial emergence of ESC-resistance in Kanagawa and in general gonococcal antimicrobial resistance in Japan have still not been completely resolved, however, they have been hypothesized elsewhere [3].

In general, during 1998–2002, the MLST ST7363 (31.6 %), ST7359 (13.9 %) and ST1901 (10.9 %) were the three most prevalent STs. During 2003–2005, the proportion of MLST ST1901 strains significantly increased, particularly among the CFM-R/DS isolates (from 2.6 % to 15.8 % of isolates). According to a recent study from 2012–2012 [24], these three MLST STs have remained the most prevalent MLST STs in the Kyoto/Osaka area; however, ST1901 has taken over as the significantly most prevalent ST (ST1901: 40.9 %, ST7359: 19.2 % and ST7363: 17.1 %). In the present study all 41 ST7359 isolates possessed NG-MAST *tbpB241* and 90.2 % contained a *porB* sequence in CL C-1, including *porB2569*, which was also the major allele among the MLST ST7359 Kyoto/Osaka strains in 2010–2012 [24]. Accordingly, MLST ST7359 strains have also been frequently isolated in many years. However, these strains have been highly susceptible to CFM and initially to also ciprofloxacin. In contrast, a major proportion of the MLST ST7363 and ST1901 strains have also been resistant to ciprofloxacin for decades. It is not evident whether CFM-R/DS acquired the ciprofloxacin-R phenotype, or vice versa, but these types of MDR strains had significant advantages, and accordingly, could rapidly and efficiently be disseminated, first locally in Japan and then globally.

## Conclusions

The two main internationally spread cefixime-resistant gonococcal clones, MLST ST7363 and ST1901 (NG-MAST ST1407 most frequent internationally) that also have shown their capacity to develop high-level ceftriaxone resistance (superbugs H041 and F89 [7, 14, 15]), likely emerged, started to disseminate and evolved in the metropolitan area, including Kanagawa, in Japan, which was followed by global transmission. A grave concern is that we might face a similar scenario in the future, i.e. that strains with resistance to both ceftriaxone and azithromycin, which are today used widely internationally in dual antimicrobial treatment regimens [31, 32], start to spread. It is crucial to understand the emergence and spread of ESC- and MDR-resistant gonococcal strains globally to develop and implement evidence-based strategies for prevention and control of gonorrhoea.

## References

[1] World Health Organization (WHO). Global incidence and prevalence of selected curable sexually transmitted infections - 2008. Geneva, Switzerland; 2012. Available at: http://www.who.int/reproductivehealth/publications/rtis/2008_STI_estimates.pdf (Accessed: May 30, 2015).

[2] Tapsall JW, Ndowa F, Lewis DA, Unemo M (2009). Meeting the public health challenge of multidrug- and extensively drug-resistant *Neisseria gonorrhoeae*. Expert Rev Anti Infect Ther.

[3] Unemo M, Nicholas RA (2012). Emergence of multidrug-resistant, extensively drug-resistant and untreatable gonorrhea. Future Microbiol.

[4] Unemo M, Shafer WM (2014). Antimicrobial resistance in *Neisseria gonorrhoeae* in the 21st Century: past, evolution, and future. Clin Microbiol Rev.

[5] Lewis DA (2010). The gonococcus fights back: is this time a knock out?. Sex Transm Infect.

[6] Ison CA (2012). Antimicrobial resistance in sexually transmitted infections in the developed world: implications for rational treatment. Curr Opin Infect Dis.

[7] Ohnishi M, Golparian D, Shimuta K, Saika T, Hoshina S, Iwasaku K (2011). Is *Neisseria gonorrhoeae* initiating a future era of untreatable gonorrhea? Detailed characterization of the first strain with high-level resistance to ceftriaxone. Antimicrob Agents Chemother.

[8] Allen VG, Mitterni L, Seah C, Rebbapragada A, Martin IE, Lee C (2013). *Neisseria gonorrhoeae* treatment failure and susceptibility to cefixime in Toronto, Canada. JAMA.

[9] Lewis DA, Sriruttan C, Müller EE, Golparian D, Gumede L, Fick D (2013). Phenotypic and genetic characterization of the first two cases of extended-spectrum cephalosporin resistant *Neisseria gonorrhoeae* infection in South Africa and association with cefixime treatment failure. J Antimicrobial Chemother.

[10] Read PJ, Limnios EA, McNulty A, Whiley D, Lahra MM (2013). One confirmed and one suspected case of pharyngeal gonorrhoea treatment failure following 500 mg ceftriaxone in Sydney. Australia Sex Health.

[11] Chen YM, Stevens K, Tideman R, Zaia A, Tomita T, Fairley CK (2013). Failure of ceftriaxone 500 mg to eradicate pharyngeal gonorrhoea. Australia J Antimicrob Chemother.

[12] Golparian D, Ohlsson A, Janson H, Lidbrink P, Richtner T, Ekelund O (2014). Four treatment failures of pharyngeal gonorrhoea with ceftriaxone (500 mg) or cefotaxime (500 mg), Sweden, 2013 and 2014. Euro Surveill.

[13] Ohnishi M, Watanabe Y, Ono E, Takahashi C, Oya H, Kuroki T (2010). Spread of a chromosomal cefixime-resistant *penA* gene among different *Neisseria gonorrhoeae* lineages. Antimicrob Agents Chemother.

[14] Unemo M, Golparian D, Nicholas R, Ohnishi M, Gallay A, Sednaoui P (2012). High-level cefixime- and ceftriaxone-resistant *N. gonorrhoeae* in France: novel *penA* mosaic allele in a successful international clone causes treatment failure. Antimicrob Agents Chemother.

[15] Cámara J, Serra J, Ayats J, Bastida T, Carnicer-Pont D, Andreu A (2012). Molecular characterization of two high-level ceftriaxone-resistant *Neisseria gonorrhoeae* isolates detected in Catalonia, Spain. J Antimicrob Chemother.

[16] Tanaka M, Nakayama H, Tunoe H, Egashira T, Kanayama A, Saika T (2002). A remarkable reduction in the susceptibility of *Neisseria gonorrhoeae* isolates to cephems and the selection of antibiotic regimens for the single-dose treatment of gonococcal infection in Japan. J Infect Chemother.

[17] Ito M, Yasuda M, Yokoi S, Ito S, Takahashi Y, Ishihara S (2004). Remarkable increase in central Japan in 2001–2002 of *Neisseria gonorrhoeae* isolates with decreased susceptibility to penicillin, tetracycline, oral cephalosporins, and fluoroquinolones. Antimicrob Agents Chemother.

[18] Deguchi T, Yasuda M, Yokoi S, Ishida K, Ito M, Ishihara S (2003). Treatment of uncomplicated gonococcal urethritis by double-dosing of 200 mg cefixime at a 6-h interval. J Infect Chemother.

[19] Yokoi S, Deguchi T, Ozawa T, Yasuda M, Ito S, Kubota Y (2007). Threat to cefixime treatment of gonorrhea. Emerg Infect Dis.

[20] Janda WM, Gaydos CA, Murray P, Baron E, Jorgensen J, Landry ML, Pfaller M (2007). *Neisseria*. Manual of Clinical Microbiology.

[21] Yamai S, Obara Y, Nikkawa T, Shimoda Y, Miyamoto Y (1979). Preservation of *Neisseria gonorrhoeae* by the gelatin-disc method. Br J Vener Dis.

[22] Clinical and Laboratory Standards Institute. 2014 Performance standards for antimicrobial susceptibility testing; 24th informational supplement. CLSI document M100-S24. Clinical and Laboratory Standards Institute, Wayne, PA, USA.

[23] Ameyama S, Onodera S, Takahata M, Minami S, Maki N, Endo K (2002). Mosaic-like structure of penicillin-binding protein 2 gene (*penA*) in clinical isolates of *Neisseria gonorrhoeae* with reduced susceptibility to cefixime. Antimicrob Agents Chemother.

[24] Shimuta K, Unemo M, Nakayama S, Morita-Ishihara T, Dorin M, Kawahata T (2013). Antimicrobial resistance and molecular typing of *Neisseria gonorrhoeae* isolates in Kyoto and Osaka, Japan in 2010–2012 – intensified surveillance after identification of the first high-level ceftriaxone resistant strain H041. Antimicrob Agents Chemother.

[25] Whiley DM, Limnios EA, Ray S, Sloots TP, Tapsall JW (2007). Diversity of *penA* alterations and subtypes in *Neisseria gonorrhoeae* strains from Sydney, Australia, that are less susceptible to ceftriaxone. Antimicrob Agents Chemother.

[26] Chisholm SA, Unemo M, Quaye N, Johansson E, Cole MJ, Ison CA, et al. Molecular epidemiological typing within the European Gonococcal Antimicrobial Resistance Surveillance Programme reveals predominance of a multidrug-resistant clone. Euro Surveill. 2013;18:ii = 20358. 23351652

[27] Lindberg R, Fredlund H, Nicholas R, Unemo M (2007). *Neisseria gonorrhoeae* isolates with reduced susceptibility to cefixime and ceftriaxone: Association with genetic polymorphisms in *penA*, *mtrR*, *porB1b*, and *ponA*. Antimicrob Agents Chemother.

[28] Whiley DM, Goire N, Lambert SB, Ray S, Limnios EA, Nissen MD (2010). Reduced susceptibility to ceftriaxone in *Neisseria gonorrhoeae* is associated with mutations G542S, P551S and P551L in the gonococcal penicillin-binding protein 2. J Antimicrob Chemother.

[29] Tomberg J, Unemo M, Davies C, Nicholas RA (2010). Molecular and structural analysis of mosaic variants of penicillin-binding protein 2 conferring decreased susceptibility to expanded-spectrum cephalosporins in *Neisseria gonorrhoeae*: role of epistatic mutations. Biochemistry.

[30] Pandori M, Barry PM, Wu A, Ren A, Whittington WL, Liska S (2009). Mosaic penicillin-binding protein 2 in *Neisseria gonorrhoeae* isolates collected in 2008 in San Francisco. California Antimicrob Agents Chemother.

[31] Bignell C, Unemo M, on behalf of the European STI Guidelines Editorial Board (2013). 2012 European guideline on the diagnosis and treatment of gonorrhoea in adults. Int J STD AIDS.

[32] Centers for Disease Control and Prevention (CDC) (2012). Update to CDC’s Sexually Transmitted Diseases Treatment Guidelines, 2010: Oral cephalosporins no longer a recommended treatment for gonococcal infections. MMWR Morb Mortal Wkly Rep.

[33] Powell AJ, Tomberg J, Deacon AM, Nicholas RA, Davies C (2009). Crystal structures of penicillin-binding protein 2 from penicillin-susceptible and -resistant strains of *Neisseria gonorrhoeae* reveal an unexpectedly subtle mechanism for antibiotic resistance. J Biol Chem.

